# Palatal Swelling: A Diagnostic Enigma

**DOI:** 10.1155/2016/1945907

**Published:** 2016-11-16

**Authors:** Ramalingam Suganya, Narasimhan Malathi, Harikrishnan Thamizhchelvan, Subramaniam Ramkumar, G. V. V. Giri

**Affiliations:** ^1^Department of Oral Pathology and Microbiology, Faculty of Dental Sciences, Sri Ramachandra University, Tamil Nadu, India; ^2^Department of Oral and Maxillofacial Surgery, Faculty of Dental Sciences, Sri Ramachandra University, Tamil Nadu, India

## Abstract

Giant cell tumor (GCT) of bone is a giant-cell-rich bony lesion associated with abundant multinucleated osteoclast-type giant cells. It is a primary neoplasm of bone with characteristic clinical, radiological, and pathological features. It is an expansive and lytic lesion without periosteal reaction and prominent peripheral sclerosis. Giant cells are also seen in other diseases like giant cell granuloma of the jaws, traumatic bone cyst, aneurysmal bone cyst, and jaw tumor of hyperparathyroidism. We present a unique case of GCT of palate in a 30-year-old female.

## 1. Introduction

Giant cell tumor of bone or Osteoclastoma is a benign giant cell tumor characterized by mononuclear cells proliferation intermixed with multinucleated osteoclast-like giant cells. However, because of their unpredictable nature, these lesions are no longer termed as “Benign.” The mononuclear cells, although considered to be nonneoplastic and reactive in nature, they are seen in distant lung metastases [1].

## 2. Case Report

**Figure 1:**
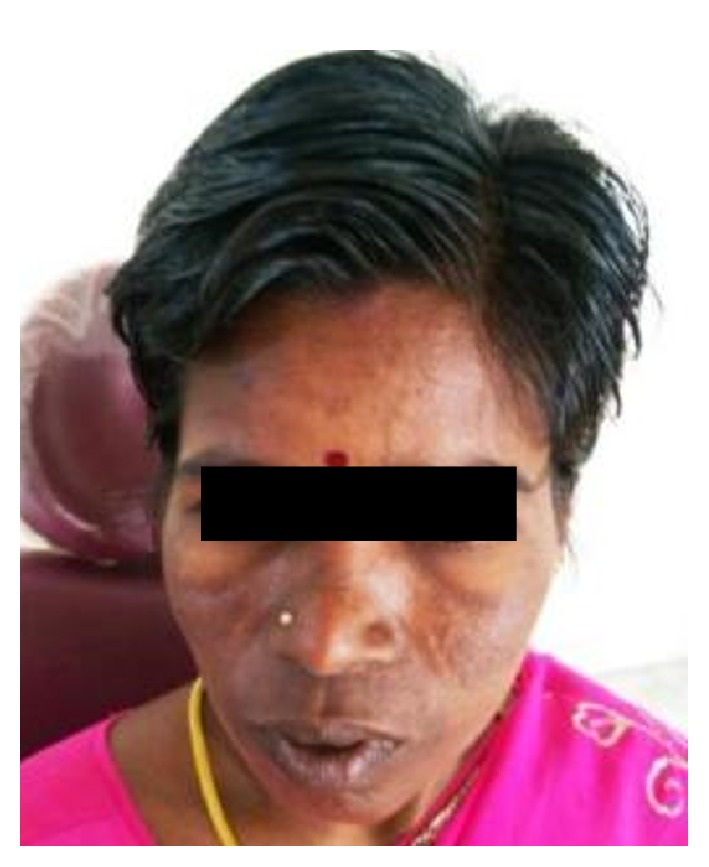
Swelling in the left maxillary region.

**Figure 2:**
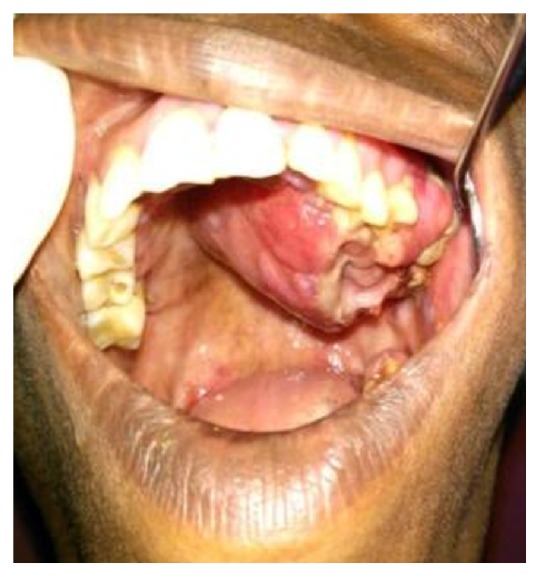
Proliferative growth of size 6 cm × 5 cm with irregular margins, extending from the 24 region up to the posterior part of the hard palate crossing the midline.

**Figure 3:**
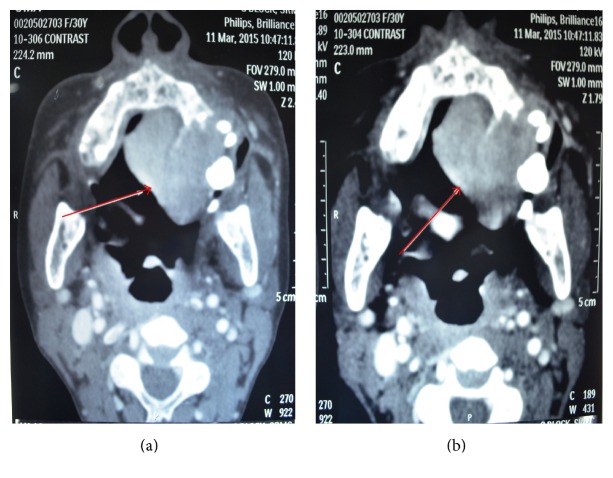
Heterogenous, well-defined, intensely enhancing lesion measuring 3 × 4.1 × 4.3 cm (cc × ap × trans) seen involving left side of buccal mucosa and the hard palate with displacement of lingual septum to right.

**Figure 4:**
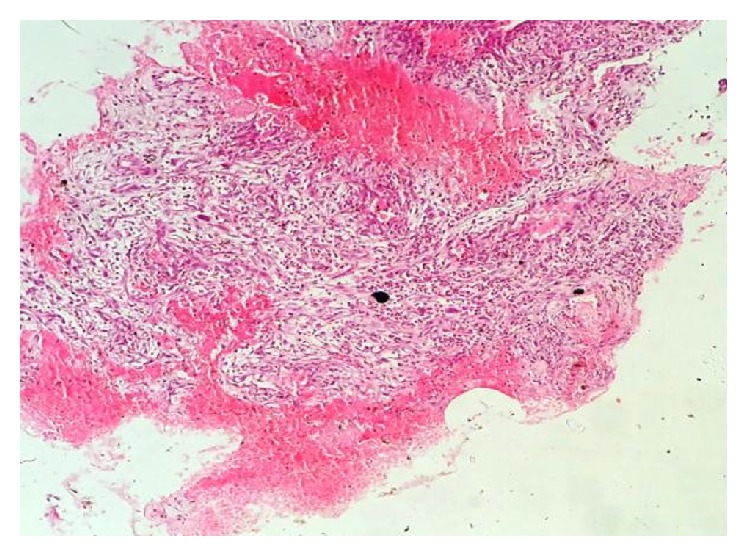
H&E 10x view showing vascular stroma with proliferation of spindle cells intermixed with extravasated RBCs.

**Figure 5:**
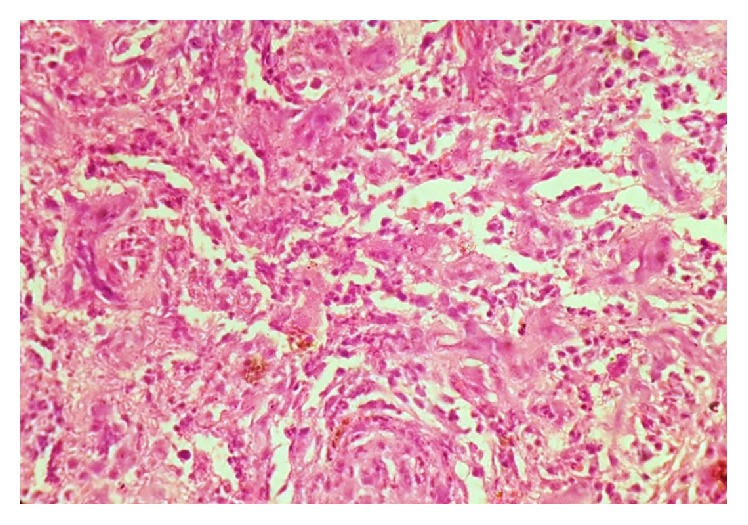
H&E 40x view showing anastomosing vascular channels lined by atypical endothelial cells.

**Figure 6:**
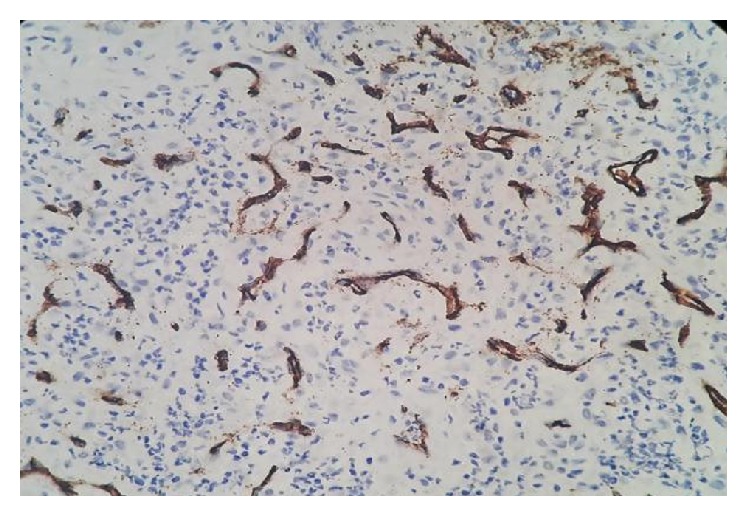
Immunohistochemical staining: showing positivity for endothelial cells to CD34 and negativity for tumor cells.

A 30-year-old female patient reported to the Department of Oral Pathology, with a swelling over the left side of the palate. Past history revealed that the patient had initially noticed the swelling 6 weeks ago. She had consulted a private dentist when the swelling was approximately 1.5 × 1.5 cm in size and had no associated symptoms (Figures 1 and 2). She was advised a biopsy, report of which revealed a histopathological diagnosis of Hemangioendothelioma. She then reported to our hospital for management of the same. On taking an elaborate history, difficulty eating and brushing was revealed. On extra oral examination, a firm swelling extending 1 cm from ala of the nose on the left side anteriorly up to 3 cm from the tragus of the left ear posteriorly was noted. On intraoral examination, a massive, solitary proliferative growth measuring 2.5 cm × 3 cm with irregular margins, extending from the left maxillary canine region up to the posterior part of the hard palate, was evident. The lesion was crossing the midline at the midpalatal region. Mucosa over the swelling was erythematous in appearance and the labial, buccal, and palatal sulci were obliterated due to buccopalatal expansion. It appeared that, at this stage, the swelling had increased in size from its initial description. Computed tomography (CT) findings revealed a heterogenous, well-defined, intensely enhancing lesion measuring 3 × 4.1 × 4.3 cm (cc × ap × trans) seen involving the left side of buccal mucosa and hard palate (Figures 3(a) and 3(b)). Laterally, an erosion of alveolar process of maxilla on left side and involvement of levator anguli oris muscle were seen, with no evidence of neovascularity. The H&E section (provided by the previous hospital of consultation) did not reveal a concrete picture of Hemangioendothelioma. An IHC analysis for CD 34 of the incisional biopsy also revealed a negativity for the tumor cells ruling out the provisional diagnosis of Hemangioendothelioma (Figures 4, 5, and 6). Based on the clinical manifestations and investigatory findings, the patient was referred to the Department of Oral and Maxillofacial Surgery for further surgical management. Partial alveolectomy of left maxillary region was planned.

Patient was placed in supine position and GA was administered. Right nasotracheal intubation was done. Considering the angiomatous nature of the lesion in maxilla, prior to Maxillectomy, the ECA was exposed and held for immediate ligation in case of untoward hemorrhage. The surgery was done as two stages: (1) neck and (2) maxilla.

Skin incision was placed on Resting Skin Tension Line on the left side of the neck, followed by layer-by-layer dissection. Weber-Ferguson incision was placed on the left side and layer-by-layer dissection done to locate the left maxillary buttress region. Osteotomy was done at Lefort I level from left pyriform aperture to maxillary tuberosity region. After complete excision of the lesion with adequate clearance, an obturator was placed over a Bismuth Iodide Paraffin Paste pack. The resected tumor was sent for histopathological examination.

**Figure 7:**
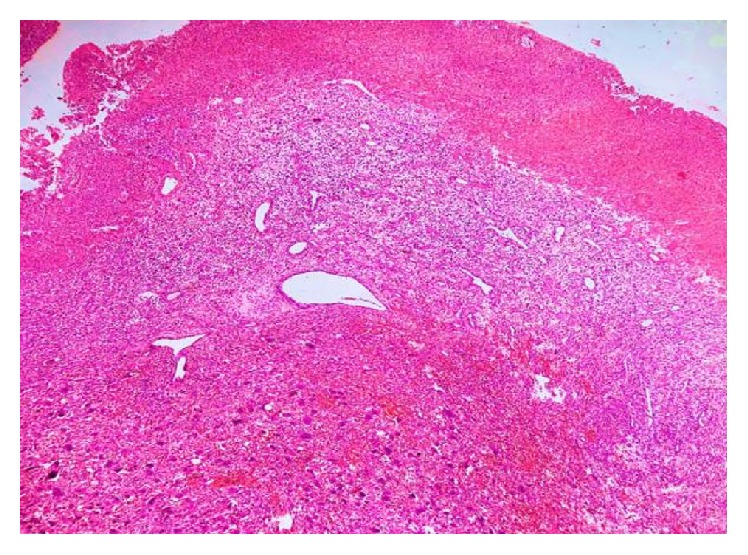
H&E 10x view vascular stroma with multinucleated giant cells.

**Figure 8:**
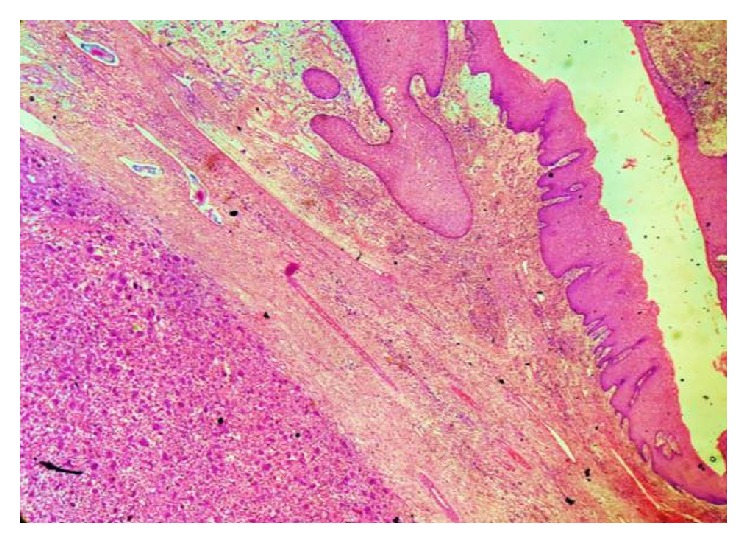
H&E 10x view overlying epithelium, connective tissue capsule, neoplastic areas showing proliferation of stromal cells, and multinucleated giant cells.

**Figure 9:**
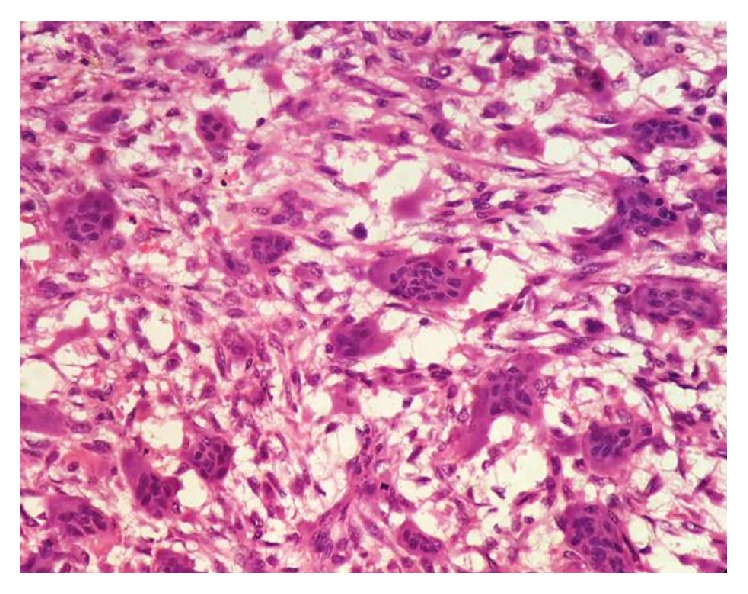
H&E 40x view showing multinucleated giant cells with agglomerate of nuclei in the center with a clear cytoplasmic halo.

**Figure 10:**
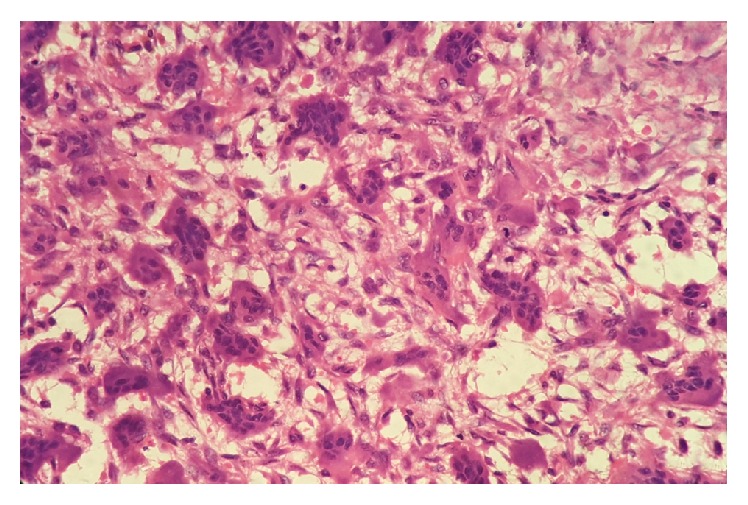
H&E 40x view showing cellular pleomorphism and mitotic activity.

Histopathological examination of the soft tissues revealed an encapsulated mass comprising stratified squamous epithelium and underlying richly cellular connective tissue stroma, containing plenty of multinucleated giant cells and dilated blood capillaries. H&E 40x view showed multinucleated giant cells with agglomeration of around 20–40 hyperchromatic nuclei in the center surrounded by clear cytoplasm and pleomorphic proliferating stromal cells. Some of the sections showed the increased vascularity with extravasation of red blood cells. Cellular pleomorphism and mitotic figures with an average of 4 per high power view were also seen which indicates local aggressiveness of this lesion (Figures 7, 8, 9, and 10). The level of serum alkaline phosphate was highly increased (320 U/L) (normal level: 45–129 U/L). A final diagnosis of giant cell tumor was given based on these characteristic findings: the characteristic appearance of proliferating stromal cells, presence of multinucleated giant cells, occurrence of cellular atypia and mitotic activity, CT, and laboratory findings. There was no evidence of recurrence in eleven months of follow-up.

## 3. Discussion

Giant cell tumor is rare and benign tumor of bone. It occurs in approximately one person per million per year [1]. Histologically, the giant cells are larger with more nuclei and evenly distributed. They may occasionally undergo malignant transformation [8]. It involves head and neck region, proximal tibia, distal femur, proximal humerus, and distal radius. Peak incidence is seen between 20 and 45 years of age [9]. Our case was reported in a 30-year-old female which involves palatal region of maxilla.

Based on clinical features and radiological and histological features, staging classification was initially proposed by Campanacci [10], which is nearly equivalent with the staging system of Enneking et al. [11]. The Enneking classification of GCT of bones is described as Stages I, II, and III and Malignant. Occurrence of obvious hemorrhage and various types of major cells like mononuclear cells of macrophage/monocyte lineage, multinucleated giant cells, and stromal cells are characteristically seen in giant cell tumor of bone [9].

Giant cell tumor of bone causes localised severe intractable epistaxis, proptosis, visual defects, hearing loss, tinnitus, reduced joint mobility, and swelling [12]. In our patient, lesion arising from palatal region of maxilla showed pain, swelling of involved region with oozing of blood, and difficulty in swallowing.

The classic radiological findings of giant cell tumor often reveal a well-circumscribed lytic lesion enclosed by minimal or no sclerosis. Tumors may break through the cortex and invade the adjacent soft tissues. A CT scan of lesion shows soft tissue mass, bony destruction, perforation of cortex, extension toward adjacent anatomic structures, resorption of teeth, and perforation of bundle bone [13]. A CT scan taken in our patient also revealed similar findings.

The appearance of gross findings of GCT of bone is variable. It is generally soft, purple-red to brown, and meaty and may be uniform or variegated in aspect, with small, spongy yellow foci or extensive areas of cystic changes [1]. In our case, grossed specimen demonstrated with blackish brown, soft to firm in consistency.

Metastasis in GCTs ranges from 1 to 6%. Lung is the main site where metastasis usually occurs [9]. Mean interval among the commencement of tumor and recognition of lung metastases is about 4-5 years [14].

The level of serum alkaline phosphate in our case was 320 U/L (normal level: 45–129 U/L). Histochemical and quantitative chemical methods show high levels of alkaline phosphate in relation to osteogenic matrix of giant cell tumor [15].

Giant cells can also be found in certain other giant cell lesions such as central giant cell granuloma, brown tumors of hyperparathyroidism, and aneurysmal bone cysts [12].

Central giant cell granuloma is a proliferative lesion, which is usually seen in young females. Clinically, it appears that destructive lesion, definite loculations and histopathological presence of proliferating spindle-shaped fibroblasts, collagen fibers, deposits of hemosiderin, patchy distribution of multinucleated giant cells, and signs of bleeding into mass are present usually on maxilla followed by mandible, whereas in our case there was no definite loculations noted [16].

Brown tumors of hyperparathyroidism show bone cysts, bone resorption, and generalized osteopenia. The most common sites are ribs, clavicle, pelvic girdle, and mandible. Deposits of hemosiderin and vascularity and presence of hemorrhage are responsible for arriving at a diagnostic terminology as “brown tumor.” Histologically, these tumors are characterized by several osteoclast-like multinucleated giant cells interspersed with infiltration of hemorrhage and deposits of hemosiderin [17].

Aneurysmal bone cysts are usually seen in vertebral column and mandible. They consist of blood filled spaces separated by fibrous septa, multinucleated giant cells, and osteoid and presence of hemosiderin and bone formation. Conventional type of ABC shows soft tissue invasion, expansive and rapid growing destructive lesion causing cortical perforation, whereas in our case absence of blood filled spaces and hemosiderin pigments were seen [18].

Treatment of GCT usually consists of intralesional curettage with autograft reconstruction [19] and wide surgical resection and placement of cement, polymethyl methacrylate [9] (Table 1).

Alcohol, hydrogen peroxide, zinc chloride, and phenol are usually applied to the lesional site. Application of hydrogen peroxide raises the infiltration of phenol into adjacent tissues. Low recurrences rate has been related to chemical adjuvants. Embolisation can be achieved by polyvinyl alcohol particles, coils, and gelfoam. Serial embolisation in large cortical effects has reduction in morbidity rate, preserve function, and relieve pain [9].

## 4. Conclusion

Various bone tumors reveal multinucleated giant cells which often should be differentiated from GCT. Early diagnosis of GCT can be done with evaluation of all the radiographic, biochemical, and histopathological limits. To attain a proper diagnosis, careful histopathological assessment is mandatory. Our case describes the difficulty in diagnosing giant cell tumors from various other lesions with which they contribute to similar behaviour, histopathology, and prognosis.

## Figures and Tables

**Table 1:** Literature review of previously reported cases of oral cavity with treatment aspects.

S. number	Authors	Year	Gender/age	Site	Follow-up	Recurrence	Treatment
1	Koszel et al. [2]	2011	17 M	Maxillary alveolar process	2 years	No recurrence	Surgical removal
2	Pradhan et al. [3]	2003	19/F	Jaw bones, orbit	Every 6 months	No recurrence	Subciliary, transperiosteal anterior orbitotomy
3	Giri et al. [4]	2015	12/F	Mandible	3 years	No recurrence	Surgical resection
4	Anand et al. [5]	2001	20/M	Hard palate	Eight months	No recurrence	Surgical excision
5	Mishra and Shukia [6]	1999	6/M	Upper alveolus, cheek	3 years	No recurrence	Surgical removal
6	Saha et al. [7]	2012	45/M	Maxilla	—	No recurrence	Partial anterolateral maxillectomy
